# Botox Therapy for Hypertrophy of the Masseter Muscle Causes a Compensatory Increase of Stiffness of Other Muscles of Masticatory Apparatus

**DOI:** 10.3390/life12060840

**Published:** 2022-06-06

**Authors:** Dorota Mierzwa, Cyprian Olchowy, Anna Olchowy, Izabela Nawrot-Hadzik, Paweł Dąbrowski, Sławomir Chobotow, Kinga Grzech-Leśniak, Paweł Kubasiewicz-Ross, Marzena Dominiak

**Affiliations:** 1 Department of Oral Surgery, Wroclaw Medical University, 50-425 Wroclaw, Poland; dorota.mierzwa@umw.edu.pl (D.M.); kinga.grzech-lesniak@umw.edu.pl (K.G.-L.); pawel.kubasiewicz-ross@umw.edu.pl (P.K.-R.); marzena.dominiak@umw.edu.pl (M.D.); 2 Department of Experimental Dentistry, Wroclaw Medical University, 50-425 Wroclaw, Poland; ania.olchowy@gmail.com; 3 Department of Pharmaceutical Biology and Botany, Wroclaw Medical University, 50-556 Wroclaw, Poland; izabela.nawrot-hadzik@umw.edu.pl; 4 Division of Normal Anatomy, Department of Human Morphology and Embryology, Wroclaw Medical University, 50-368 Wroclaw, Poland; pawel.dabrowski@umw.edu.pl; 5 Department of General Radiology, Provincial Specialist Hospital in Legnica, 59-220 Legnica, Poland; slavek1@gmail.com

**Keywords:** masseter muscle, temporalis muscle, shear-wave elastography, stiffness, muscle hypertrophy

## Abstract

Little is known about the nature of masseter muscle hypertrophy. We investigated the masseter muscle stiffness change after a single intra-masseteric session of Botox injections in people with benign bilateral masseter hypertrophy and the effect of the treatment on the stiffness of the temporalis muscle. Stiffness of the muscles was measured with shear-wave elastography at baseline and 3 weeks after Botox injections in 22 otherwise healthy people. Before the treatment, the stiffness of the masseter was lower than of the temporalis muscle (10.18 ± 1.67 kPa vs. 11.59 ± 1.54 kPa; *p* = 0.002). After the treatment, this difference increased (6.38 ± 1.34 vs. 13.10 ± 1.92; *p* < 0.0001). The drop in the stiffness of the masseter muscle was symmetrical (left side by 3.78 kPa; right side by 3.83 kPa). No differences between the left and right sides of the face in terms of muscle stiffness were observed. The study shows that Botox injections reduce stiffness of the masticatory muscles which, in turn, increases the stiffness of the temporalis muscles. Due to the knock-on effect of the change in the masseter function on the other masticatory muscles, simultaneous evaluation and treatment of the temporalis muscle may be required to ensure the desired functional and cosmetic effect.

## 1. Introduction

Little is known about the nature and characteristics of masseter muscle hypertrophy. It can affect one side or both sides of the face and is characterized by an increase in the volume of the muscle tissue, resulting in the change in the contour of the face. In the literature, masseter muscle hypertrophy is often described as a concomitant condition to the hypertrophy of other masticatory muscles, as this condition affects masseteric and temporalis muscles [1] or even medial pterygoid hypertrophy [2]. Patients with unilateral masseter muscle hypertrophy are often consulted by an esthetic surgeon due to face asymmetry [3,4]. Bilateral enlargement of the masseter muscles is more common, but constitutes a smaller esthetic problem maintaining the symmetry of the face [1]; however, it is more often associated with other symptoms, such as bruxism [2,5], temporomandibular disorders, malocclusion, pain, otalgia, nighttime trismus [6], emotional stress [1,3,6], and oral parafunctions, including unilateral chewing and excessive chewing gum [2,7]. Furthermore, together with the increased muscle mass, changes in the adjacent bone tissue may occur, such as a thickened cortex of the angle and ramus of the mandible, temporal fossa, and zygomatic arch with a corresponding decrease in marrow area or prominent exostoses at the angle of the mandible, visible in the CT scans [3,5]. 

Idiopathic etiology is common among patients with masseter muscle hypertrophy [6]. Some researchers divide this condition into congenital and acquired [8], while others consider all cases congenital with a variable presentation [9]. Unilateral enlargement of the masseter muscles is less frequently prevalent and maybe associated with other pathologies [10,11]. Although the exact etiology of acquired masseter muscle hypertrophy is not known, there are several factors associated with its development, such as bruxism, oral parafunctions, or malocclusions [2,5,7], but it is not clear whether they are causative or resultant factors. As shown in previous research, intensive chewing gum can increase the stiffness of the masseter muscle [12] and lead to bilateral masseter hypertrophy [7]. Most patients report slow but progressive nature of the condition [2,3,5,13]. The muscle tone pathophysiology in patients with masseter muscle hypertrophy has not been investigated. However, sensory biofeedback may play a role in its development. Due to the lack thereof, the patient may have difficulties with optimizing movements with a subsequent increase in muscle contraction around the joints [14].

Epidemiology data on masseter muscle hypertrophy are limited. Rispoli et al. mention a review of 108 cases which found that affected patients were 30 years old on average, 57% were males, 60% had bilateral pathology, and only 5% had concurrent temporalis muscle hypertrophy [6]. Masseter muscle hypertrophy is rare in children [15]. The incidence of masseter muscle hypertrophy is considered higher in Asians who are, at the same time, more sensitive about their lower face contouring [4].

Patients usually seek medical advice because of dissatisfaction with their appearance; however, some of them experience problems caused by hypertrophy that include chronic pain of the frontal and temporal area of the head [1,7]. Treatment includes surgery and botulinum toxin type A (Botox) injections [16,17]. Botox injections are common and may have some advantages over surgical treatment. Despite effectiveness of Botox injections in people with bilateral masseter hypertrophy being well documented, none of the studies uses objective methods to document and monitor treatment effects, such as shear wave elastography (SWE).

Measurement of stiffness of masticatory muscles using SWE is gaining attention in dentistry [18]. Elastography is a type of imaging technique utilizing a probe that generates mechanical vibration resulting in propagation of low-frequency shear waves inside tissues, creating two intense plane shear waves [19]. These shear waves distort soft tissues to a degree corresponding to their stiffness. Next, they are detected and registered by an ultrafast scanner. The stiffness of the tissues can be mapped quantitatively from this propagation image by inversion algorithms; thus, this technique allows determining the actual elastic modulus of soft tissues in the set region of interest (ROI). SWE can quantitatively record tissue stiffness in kPa. To date, normal values of the stiffness of the masseter muscle have already been established [20,21]. This study further investigates stiffness of the masseter muscle in pathological conditions. In the present study, we investigated a degree of a reduction in the masseter muscle stiffness after a single intra-masseteric session of Botox injections in people with benign bilateral masseter hypertrophy. Furthermore, we intended to investigate whether a drop of the masseter stiffness would be bilaterally equal. Symmetry of the injection grid should be crucial to ensure satisfactory aesthetic outcomes [22]; however, in daily practice, injecting the medication into five mirror sites of both masseters and equally dividing the dose into those five injections still poses a challenge. Another interesting issue is the potential influence of iatrogenic reduction in the masseter muscle stiffness on other muscles of the masticatory apparatus; however, hypertrophy of the temporalis muscles is considered an esthetic problem, particularly when hypertrophy is only unilateral [2,23,24]. The pterygoid muscles are not easily accessible for the ultrasound and elastography examination, therefore, we focused on the temporalis muscles. 

## 2. Materials and Methods

### 2.1. Participants

We enrolled adults (individuals over 18 years of age) with idiopathic bilateral masseter muscle hypertrophy. All participants gave their informed consent for participation in the study. Patients treated previously with Botox injections for masseter muscle hypertrophy were not considered. This longitudinal single-arm study was conducted according to the Declaration of Helsinki and was approved by the Bioethical Committee at Wroclaw Medical University (KB—633/2020).

Exclusion criteria were as follows: concomitant pathological conditions affecting masseter muscles such as temporomandibular disorders (evaluated based on the Diagnostic Criteria for Temporomandibular Disorders protocol [25]), neuromuscular disorders and inflammatory diseases of this region, prior surgery for masseter muscle hypertrophy, the presence of malignant or benign lesions of this area, pain within masseter muscles, parafunctional oral habits reported by the patients, systemic diseases treated with drugs affecting muscle functioning as well as breastfeeding and pregnancy.

### 2.2. Study Procedure

**Figure 1:**
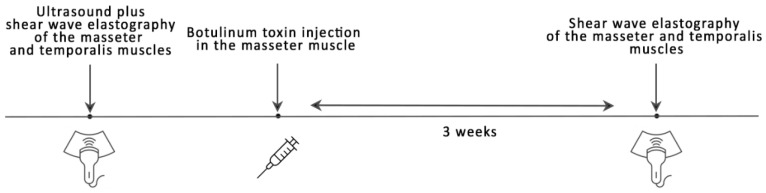
Study procedure.

Before SWE measurement, an ultrasound examination of both masseter muscles was performed to exclude any local occult pathologies, such as parotid enlargement. The study protocol was based on the experience of the Kim’s team [26], who reported the use of Botox in a large group of patients with masseter muscle hypertrophy. Immediately after SWE, a Botox injection was administered. The next SWE was performed after 3 weeks. We also validated the measurements against the values obtained from an elasticity QA Phantom model 049A (Computerized Imaging Reference Systems, Inc, Norfolk, VA, USA). The study design is schematically shown in Figure 1.

### 2.3. Botox Treatment

For the Botox treatment, BOTOX^®^ Cosmetic (onabotulinumtoxinA, Allergan USA Inc., Madison, NJ, USA) was used. Each patient received three or four injections (20–25 Ul in total) into each masseter muscle depending on the degree of his/her muscle hypertrophy. Injections were made manually and based on the operator experience. Injections were planned to be symmetrical on both sides of the face due to the fact that all participants suffered from bilateral masseter muscle hypertrophy.

Botox injections were conducted with caution and with consideration of anatomical structures of the masseter muscle. Patients who received four injections were injected according to the Kim et al. method [26]. Patients who received three injections were injected according to the modified method developed in our institution. The principles of the methods are as follows: the effect of Botox is 1 cm; the puncture area is marked by three intersecting lines creating a safe area 15–20 mm above the edge of the mandible; and the drug is divided into three parts in a 2:1:1 ratio, with the highest dose at the highest point.

### 2.4. Shear Wave Elastography

**Figure 2:**
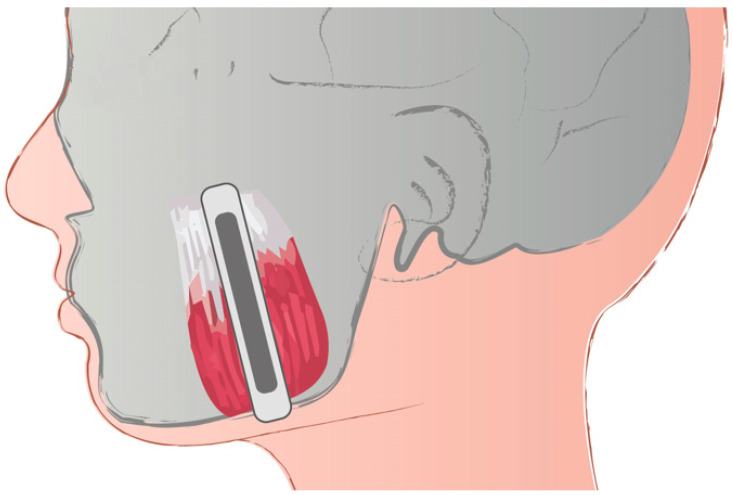
Placement of the probe on the masseter muscle during shear wave elastography examination (reproduced from Olchowy et al. [20]).

Qualified participants were referred for the SWE examination. The methodology of SWE was described elsewhere [27]. Briefly, the probe was placed parallel to the longitudinal axis of the masseter muscle in the middle part of the muscle in the belly. The middle of the masseter muscle was identified as the widest part of the muscle during clenching as the most protruding fragment of the masseter muscle. On the temporalis muscle, the probe was placed on the muscle just above the zygomatic arch and parallel to the fibers of the temporalis muscle. Our previous experience shows that such placement of the probe allows for easy identification of the target area and for obtaining accurate and repeatable measurements (Figure 2). An ROI of 4-mm was used. Participants were asked to lie in a relaxed, supine position and refrain from swallowing.

The SWE measurements of the masseter muscle were taken three times; an averaged result was analyzed. We used the Aixplorer Ultimate device (SuperSonic Imagine, Aix-en-Provence, France) using a high-frequency linear probe SL 18-5 (5–18 MHz). Shear waves propagate in the tissues with a speed of 1 m/s–10 m/s which corresponds to elasticity of 1–300 kPa. We validated the measurements with the elasticity QA Phantom model 049A (Computerized Imaging Reference Systems, Inc., Nor-folk, VA, USA). All examinations were performed by a radiologist with eight years of experience in shear wave elastography, including experience in masticatory muscles evaluation.

### 2.5. Statistical Analysis

Statistical analysis was carried out with MedCalc v. 19.5.3 (MedCalc Software Ltd., Ostend, Belgium). The Shapiro–Wilk test was used to check for the normality of the data. Means with standard deviations were calculated. Normality was checked and was rejected for all variables. The Mann–Whitney U was used to compare independent samples and the Wilcoxon test was used for repeated measures. A probability value lower than 0.05 was considered statistically significant.

## 3. Results

The study included 23 people with an average age of 49 ± 8 years (15 women and 8 men). One woman was excluded because she developed an inflammatory condition of the molar with a denticle.

**Figure 3:**
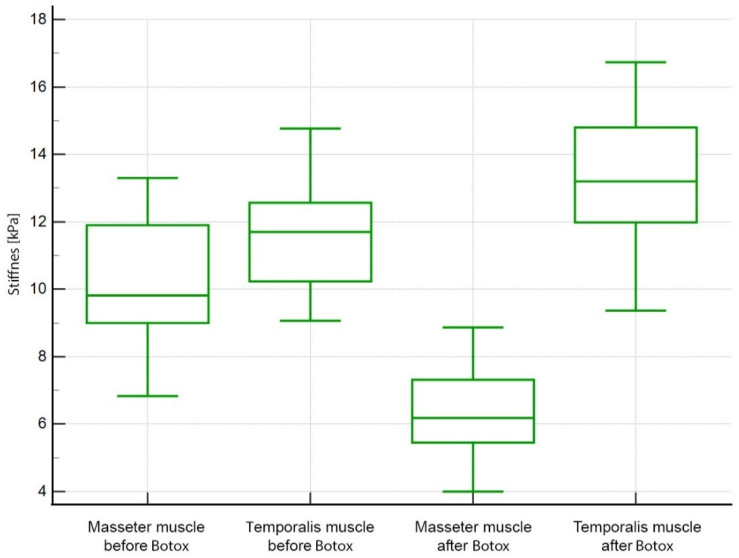
Comparison of the stiffness before and after the treatment with Botox.

The comparison between the overall muscle stiffness before and after Botox injections is shown in Figure 3. Before the treatment, the overall stiffness of the masseter muscle was significantly lower than the overall stiffness of the temporalis muscle (10.18 ± 1.67 kPa vs. 11.59 ± 1.54 kPa; *p* = 0.002). The difference in stiffness between the muscles was greater after the treatment with Botox (6.38 ± 1.34 vs. 13.10 ± 1.92; *p* < 0.0001). The interaction effect (difference of differences) for the masseter muscle was 3.80 kPa and for the temporalis muscle −1.55 kPa (*p* < 0.001). Furthermore, the drop in the stiffness of the masseter muscle was similar (symmetrical) on both sides. It was 3.78 kPa for the left side and 3.83 kPa for the right side. No differences between the left and right sides of the face in terms of muscle stiffness were observed. Detailed comparisons are shown in Table 1.

## 4. Discussion

Our study showed a significant drop in the stiffness of the masseter muscle after 3 weeks following Botox injection with a parallel increase in the stiffness of the temporalis muscle. Patients were satisfied with the physical appearance of the lower contour of the face as a result of the reduction of muscle volume; however, we did not use any cosmetic scale to make a quantitative measure. All patients included in the study suffered from bilateral masseter muscle hypertrophy. This is well reflected in the stiffness results as the obtained results are symmetrical with an almost equal drop in stiffness on both sides (by 3.78 kPa for the left masseter and by 3.83 kPa for the right masseter). Regarding the masseter muscle, the overall stiffness of the hypertrophic masseter muscle was 10.18 ± 1.67 kPa in comparison to our previously conducted studies on healthy adults where the stiffness of the masseter muscle ranged from 10.54 kPa to 11.46 kPa [12,18,27].

Botox injections into masseter muscle tissue for masseter muscle hypertrophy have been used since 1994, since Smyth described such treatment in seven patients with bilateral masseter muscle hypertrophy [28]. A systematic review on the use of Botox for bilateral benign masseter hypertrophy conducted by Fedorowicz et al. in 2013 stated that the evidence on the efficacy and safety of intra-masseteric injections of Botox was still insufficient to draw reliable conclusions [17]. However, recent reports confirm advances in this field and present a tailored approach which increases effectiveness and reduces complication rate [29]. In a randomized controlled study conducted by Hong et al. (2021), the effective dose range for a reduction of the masseter muscle ranged from 48 to 72 units. They also found that gradual reduction was observed up until 12 weeks and after that period, reinjection should be considered for maintaining satisfactory cosmetic effect [30].

Up until now, the effectiveness of the treatment with Botox is assessed using several tools. The most common is ultrasound, used for measuring muscle thickness. It also helps to evaluate the condition of the masseter muscle and surrounding tissues, thus, differential diagnosis. Ultrasound measures muscle thickness which is helpful for the determination of the optimal dose of Botox [26,30,31,32,33]. Magnetic resonance and computerized tomography of the masseter regions are performed commonly for the purpose of differential diagnosis with some attempts to use them for assessments of treatment effects [34]. Apart from the condition of the masseter muscle, it can provide information on the structure and shape of the mandible, and help to evaluate parotid and submandibular glands [2,5,31]. Many physicians make standardized photographs with the muscles relaxed and with patients grinding or serial photographs to document the effects of treatment [26,33,35]. Monitoring and documenting the effects of treatment is particularly important because there is only one scale to classify masseteric hypertrophy and cosmetic results of its treatment [4]. Shear wave elastography has not been used for this purpose. However, apart from monitoring the desired decrease in muscle tonus, is can be used for evaluation of side effects such as fatigue and muscle weakness (associated with too much drop in muscle tonus) which can be associated with inadequately tailored Botox dose [36]. There are ongoing projects on the determination of desired values of masseter muscle stiffness as measured by shear wave elastography [37]. Advantages of shear wave elastography include simplicity, reliability, and repeatability. It can objectively measure tissue and organ stiffness. It is characterized by good diagnostic accuracy in measuring masseter muscle stiffness [18]. It is also found to be a reliable and repeatable method for the assessment of muscle stiffness [38]. SWE proved to be a sensitive tool for monitoring changes of masseter muscle stiffness so that it can help identify treatment failure or asymmetry occurring during Botox treatment due to inadequate dose of Botox, odontogenic inflammation, or misdiagnosis. Previous studies showed that masseter muscle stiffness changes after treatment or exercise regardless of baseline values [12,27] which makes it particularly useful for evaluation of conducted treatment. Furthermore, it can be used by physicians of all specialties [18].

Besides the masseter muscle, hypertrophy of masticatory muscles may involve the temporalis muscle and medial pterygoid muscle as well. Enlargement of other muscles is usually observed at the diagnosis. Guruprasad et al. reported coexistent enlargement of the medial pterygoid muscle in addition to the masseter muscle hypertrophy, which is rare [2]. Graziano et al. treated a series of patients some of which had unilateral or bilateral masseteric and temporalis muscle hypertrophy. Such patients received Botox injections into both affected muscles [1]. The present study included patients with isolated hypertrophy of the masseter muscle. Any other pathologies of adjacent muscles were not seen.

During Botox treatment for masseter muscle hypertrophy, enlargement of other masticatory muscles may occur because the temporalis muscle can take over the functions of the paralyzed muscles lifting the mandibula. In the present study, we observed that the temporalis muscle became slightly enlarged on the second follow-up visit after treatment with Botox. Furthermore, a compensatory increase in the temporal muscle stiffness (*p* < 0.005 for both sides) was observed; however, the differences in terms of the magnitude of the stiffness increase were observed among patients. These differences may be due to many factors, including chewing patterns and diet. Preliminary reports from the literature suggest that different foods exert differentiated effect on the activity of the masseter and temporalis muscle [39]; further research is required in this scope. It can also be hypothesized that the long-term increase in tonus of the temporalis muscle (some patients undergo a Botox treatment repeatedly) may result in hypertrophy of the temporalis muscle belly and, as a result, a change in the proportion/oval of the face. Perhaps, after identifying the factors influencing a greater increase in the temporalis muscle stiffness in subsequent studies, administration of Botox should be considered in patients with risk factors for the temporalis muscle hypertrophy. It may also be possible that all patients with masseter muscle hypertrophy should be subjected to a simultaneous Botox treatment of the temporalis muscle. The Botox treatment of the masseter muscle affects remaining masticatory muscles as well, but they are not easy to evaluate with shear-wave elastography as the medial pterygoid muscle or play a little role in the lifting of the jaw as the lateral pterygoid muscle. For these reasons, the impact of Botox injections into the masseter muscle is difficult to assess. However, these muscles have a relatively small volume and, therefore, little strength, and have no effect on the face oval. Given the above, it should be noted that the masticatory system works like a system of communicating vessels and treatment of a single pathology may disturb the balance resulting in the development of another pathology. For this reason, careful assessment of all masticatory muscles is recommended during long-term treatment to ensure that the entire contour of the face is satisfactory to the patient. Using shear wave elastography during follow-up visits, we are able to detect increasing stiffness of other muscles of the masticatory system even before the muscle enlargement is visible and palpable.

## 5. Limitations

Several limitations of this study should be mentioned. The study group included a small number of people with a single pathology. For this reason, it was relatively homogeneous, which did not allow investigation of the relationships among all masticatory muscles. Next, we did not use any other tool to assess the muscle condition or cosmetic effect achieved by treatment. However, the overall evidence on masseteric hypertrophy is limited as is the availability of other methods. In the future studies, researchers should focus on the use of safe and convenient methods such as shear wave elastography and validate them against previously used objective methods which may expose the patient to radiation or contrast use such as panoramic radiography, computed tomography, magnetic resonance imaging. Furthermore, the reference values of masticatory muscles have not been established yet, so we are able to monitor stiffness in one patient on subsequent visits. Although several studies were published on the use of shear wave elastography for measuring the masseter muscle stiffness [12,18,27,37], other muscles of the masticatory system still need to be investigated. Finally, shear wave elastography can give information only on the muscle stiffness without explaining physiology of the muscle tone. For this reason, it cannot be used to investigate the causes of masseter muscle hypertrophy that could be useful for the development of tailored treatments.

## 6. Conclusions

This study allowed for drawing several valuable conclusions. First, a drop in the stiffness of the masseter muscles after a single session of Botox injections was symmetrical despite the manual division of the application between 3–4 injection sites. This indicates that some discrepancies are acceptable. It also suggests that the effectiveness of the dose in each patient simply means that a certain field of work on dose reduction in subsequent studies is open, so as to determine the lowest effective dose in all patients. Second, the function of the masseter muscles, as a part of the whole masticatory muscle system, may have an effect on other masticatory muscles. The study shows that an iatrogenic reduction of the masseter muscle stiffness results in a compensatory increase in the temporal muscle stiffness (*p* < 0.005 for both sides). For this reason, a simultaneous Botox treatment of the temporalis muscle may be required in all or selected patients undergoing treatment for masseter hypertrophy. However, further research is needed to investigate the role of risk factors for temporalis hypertrophy.

Shear wave elastography can help to monitor treatment effect with Botox of masseter muscles in muscle hypertrophy and other conditions (e.g., TMD, bruxism). In comparison to currently used methods, it offers additional benefits for the patient, such as better dose adjustment and prompt information about the condition of other non-treated masticatory muscles. This preliminary study shows the potential of shear wave elastography, but further research is required to gain experience and determine stiffness values of hypertrophic masticatory muscles. Furthermore, studies on muscle physiology can help determine the usefulness of shear wave elastography. 

The study should be considered preliminary. Further research is needed on larger groups of patients and with longer follow-up to fully evaluate the effectiveness of the proposed treatment and changes in the muscle stiffness. 

## Figures and Tables

**Table 1:** Elasticity values before and after massage (data are shown in kPa).

N = 23	Before Botox	After Botox	*p*-Value
Masseter muscle			
Left masseter	10.16 ± 1.74	6.38 ± 1.36	<0.0001
Right masseter	10.20 ± 1.65	6.37 ± 1.34	<0.0001
*p*-value	0.6302	1.000	
Temporalis muscle			
Left temporalis	11.57 ± 1.55	13.12 ± 1.96	0.0002
Right temporalis	11.62 ± 1.57	13.09 ± 1.92	0.0013
*p*-value	0.9813	0.9813	

## Data Availability

The data presented in this study are openly available in FigShare.
